# A Review of Imaging Techniques for Plant Phenotyping

**DOI:** 10.3390/s141120078

**Published:** 2014-10-24

**Authors:** Lei Li, Qin Zhang, Danfeng Huang

**Affiliations:** 1 School of Mechanical Engineering, Shanghai Jiaotong University, Shanghai 200240, China; E-Mail: hudiepianpianlilei@hotmail.com; 2 Center for Precision & Automated Agricultural Systems, Washington State University, 24106 N. Bunn Rd., Prosser, WA 99350, USA; E-Mail: qinzhang@wsu.edu; 3 School of Agriculture and Biology, Shanghai Jiaotong University, Shanghai 200240, China

**Keywords:** phenotyping phenotype, fluorescence imaging, thermal infrared imaging, visible light imaging, imaging spectroscopy, three dimensional imaging

## Abstract

Given the rapid development of plant genomic technologies, a lack of access to plant phenotyping capabilities limits our ability to dissect the genetics of quantitative traits. Effective, high-throughput phenotyping platforms have recently been developed to solve this problem. In high-throughput phenotyping platforms, a variety of imaging methodologies are being used to collect data for quantitative studies of complex traits related to the growth, yield and adaptation to biotic or abiotic stress (disease, insects, drought and salinity). These imaging techniques include visible imaging (machine vision), imaging spectroscopy (multispectral and hyperspectral remote sensing), thermal infrared imaging, fluorescence imaging, 3D imaging and tomographic imaging (MRT, PET and CT). This paper presents a brief review on these imaging techniques and their applications in plant phenotyping. The features used to apply these imaging techniques to plant phenotyping are described and discussed in this review.

## 1. Introduction

To ensure that crop production is sufficient to satisfy the needs of a human population that is expected to grow to more than 9 billion by 2050 is a tremendous challenge for plant science and crop improvement [1]. This goal is challenging primarily because the average rate of crop production increase is only 1.3% per year, and it cannot keep pace with population growth. By connecting the genotype to the phenotype, high yielding, stress-tolerant plants can be selected far more rapidly and efficiently than is currently possible. Advances in techniques such as next generation DNA sequencing can be made available to breeders to provide potential increases in the rate of genetic improvement by molecular breeding [2]. However, the lack of access to phenotyping capabilities limits our ability to dissect the genetics of quantitative traits related to growth, yield and adaptation to stress. Plant breeders and farmers were making selections based on phenotypes long before the discovery of DNA and molecular markers. To identify the best genetic variation, the more crosses and environments that are used for selection, the greater the probability of identifying a superior variation. To meet future requirements, there is a need to increase breeding efficiency. Advances in high throughput genotyping have offered fast and inexpensive genomic information and paved the way for the development of large mapping populations and diversity panels of thousands of recombinant inbred lines for phenotyping [3]. Although molecular breeding strategies have placed greater focus on selections based on genotypic information, they still require the following phenotypic data [4]: (1) phenotypes are used for selection and to train a prediction model in genomic selection; (2) a single phenotyping cycle is used to identify markers for subsequent selection through generations within the maker-assisted recurrent selection [5]; and (3) phenotyping is necessary to identify promising events in transgenic studies [6]. Phenotyping advances are essential for capitalizing on developments in conventional, molecular, and transgenic breeding.

### 1.1. Plant Phenotyping

Plant phenotyping is the comprehensive assessment of complex plant traits such as growth, development, tolerance, resistance, architecture, physiology, ecology, yield, and the basic measurement of individual quantitative parameters that form the basis for more complex traits [7]. The plant phenotype includes these complex traits, and examples of their direct measurement parameters are the root morphology [8–11], biomass [12,13], leaf characteristics [14,15], fruit characteristics [16,17], yield-related traits [18], photosynthetic efficiency [19], and biotic and abiotic stress response [20,21]. Given the rapid development of high-throughput genotype screening in plant breeding and genomics for related growth, yield and tolerance to different biotic and abiotic stresses, there is a call for more effective and reliable phenotyping data to support modern genetic crop improvement. Current assessments of phenotype characteristics for disease resistance or stress in breeding programs rely largely on visual scoring by experts, which is time-consuming and can generate bias between different experts and experimental repeats. Plant phenotyping has become a major field of research in plant breeding [22]. Plant phenotyping is intended to measure complex traits related to growth, yield and adaptation to stress with a certain accuracy and precision at different scales of organization, from organs to canopies [23]. To accomplish this goal, phenotyping enlists expertise from the biological sciences, computer science, mathematics and engineering. In recent years, high throughput phenotyping platforms have been deployed in growth chambers or greenhouses [24–26]. These platforms use robotics, precise environmental control and imaging technologies (hardware and software) to assess plant growth and performance. However, these platforms are designed for a limited range of species, encompassing small rosette plants such as *Arabidopsis* [14,15,27] and the primary cereal crops [13,28–30]. Generic platforms and solutions enabling the simultaneous phenotypic evaluation of multiple species must be developed.

### 1.2. The Role of Imaging Techniques in Plant Phenotyping

**Figure 1.**
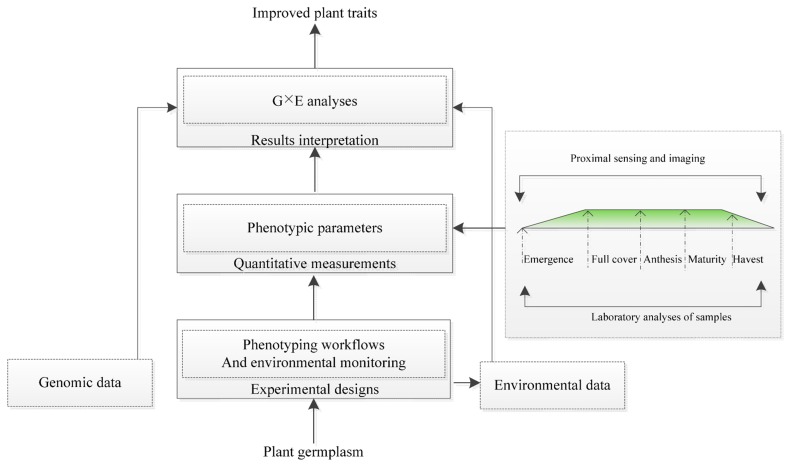
A scheme for plant phenotyping [31,34].

To analyze gene-environment (G × E) interactions and model phenotypic responses, the scheme for plant phenotyping usually includes an experimental design, quantitative measurement and results interpretations (Figure 1). The experimental design must consider different growth environments (a controlled environment or a field). It also simultaneously includes the plant growth infrastructure, environmental monitoring, substrate handling and biosafety installations. Quantitative measurement strongly benefits from novel imaging technologies but needs standardized experimental protocols, including imaging sensor calibration and a precise definition of raw data processing routines, as part of the best practices for plant phenotyping. Results interpretation requires the integration of experimental metadata within data schemas for the measured phenotype, genomic data and environmental data [23,31]. Quantifying the plant phenotype is a key step for implementing plant phenotyping. Modern imaging techniques have high resolution and allow for the visualization of multi-dimensional and multi-parameter data. Imaging techniques are used to quantify complex traits under related growth, yield and applications to stress for plant phenotyping in controlled environmental systems (in growth chambers or in the greenhouse) or in the field [15,31–33]. The use of imaging techniques to monitor plant growth and dynamic responses under stress in real time can also be more readily achieved.

Image analysis algorithms are the primary drivers for advancing imaging-based studies that require the quantification of plant phenotypes for parts such as the roots, stems, leaves, seeds, and flowers. Ninety-two different image analysis software tools were described on a website for studying plant biology [35]. Some of these tools require user inputs such as manual point selection, whereas others are automated or semi-automated. Typical segmentation algorithms are based on a color model and threshold value and could evaluate the plant growth and rosette geometry time courses by extracting the projected shoot area and geometric parameters in a 2D RGB image [36,37]. Feature extraction and image analysis software for the 2D and 3D analysis of shoot and root growth and architecture are useful for plant phenotyping [37–39]. Based on images of the phenotype, quantitative measurements for complex traits under related growth, yield and applications to stress primarily rely on imaging processing algorithms.

Imaging plants is more than just ‘taking pictures’. The aim of imaging is to measure a phenotype quantitatively through the interaction between light and plants such as reflected photons, absorbed photons, or transmitted photons. Each component of plant cells and tissues has wavelength-specific absorbance, reflectance, and transmittance properties. For example, chlorophyll absorbs photons primarily in the blue and red spectral region of visible light; water has its primary absorption features in the near and short wavelengths and cellulose absorbs photons in a broad region between 2200 and 2500 nm. Imaging at different wavelengths is used for different aspects of plant phenotyping (shown in Table 1). Visible imaging is primarily used to measure aspects of plant architecture such as image-based projected biomass, leaf area, color, growth dynamics, seedling vigor, seed morphology, root architecture, leaf disease severity assessments, yield, and fruit number and distribution. Fluorescence imaging was used for disease detection in genetic disease resistance. Thermal infrared imaging could characterize the plant temperature to detect differences in stomatal conductance as a measure of the plant response to the water status and transpiration rate for abiotic stress adaptation. Imaging spectroscopy can provide insight into the drivers of growth dynamics by means such as measuring spatiotemporal growth patterns during experiments and also for gathering plant spectroscopy data to quantify vegetation indices, water contents, the composition parameters of seeds and pigment composition in yield potential studies. At present, imaging techniques for plant phenotyping primarily include fluorescence imaging, thermal infrared imaging, visible imaging, imaging spectroscopy and other techniques (MRI, PET and CT). With regards to the organization of this review paper, each imaging technique is profiled with its respective underlying principle, a description of selected current applications, and a discussion of advantages and known limitations in plant phenotyping.

## 2. Key Imaging Techniques in Plant Phenotyping

**Figure 2.**
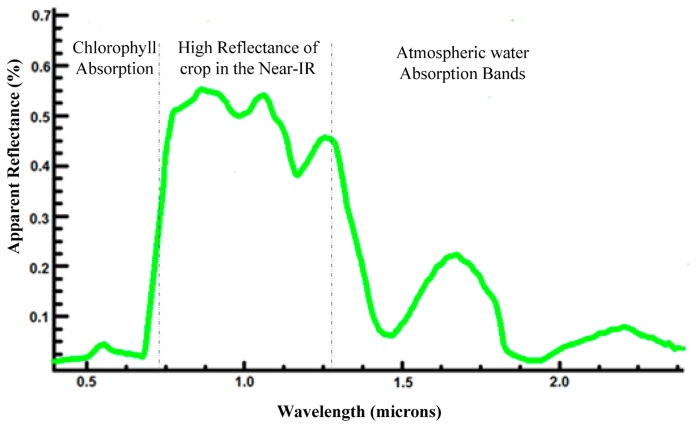
The typical reflectance spectra of crop at different wavebands [92].

Detection information carriers are considered to be electromagnetic waves. Healthy plants interact (absorb, reflect, emit, transmit and fluoresce) with electromagnetic radiation in a manner different from that of infected plant interactions (as shown in Tables 1 and 2). This finding is primarily explained by the fact that plants have different optical properties. Imaging techniques are very helpful for detecting these properties, especially for those that cannot be seen by the naked eye. Plant phenotyping based on spectral reflection information relies on the properties of the light emerging from the canopy after multiple interactions (such as reflections, transmissions, and absorptions) with the tissues of the plant. The canopy spectral signature from this diffusely reflected radiation is described by the ratio of the intensity of reflected light to that of the illuminated light for each wavelength in visible (400–750 nm), near-infrared (750–1200 nm) and shortwave infrared (1200–2400 nm) spectral regions. Leaf reflectance is defined as the proportion of the irradiated light that is reflected by the leaf. The leaf interaction of electromagnetic radiation with plants varies with the wavelength of the radiation. Because of the strong absorption by photoactive pigments (chlorophylls, anthocyanins, and carotenoids) at visible wavelengths, the canopy has low reflectance. In the near-infrared wavebands, the canopy has high reflectance because of multiple scattering at the air-cell interfaces in the internal leaf tissue. In wide wavebands of shortwave infrared, healthy leaves have low reflectance because of absorption by water, proteins and other carbon constituents. The typical reflectance spectra of crops in these three wavebands are shown in Figure 2. Because of their high water content (emissivity between 0.97 and 0.99), healthy leaves emit radiation in the thermal infrared band (≈10 μm) according to their temperature. The leaves appear green because the green light band (550 nm) is reflected relatively efficiently when compared with the blue, yellow and red bands, which are absorbed by photoactive pigments. At approximately 670 nm, reflectance changes cause the red edge to shift to shorter wavelengths (the sharp transition from low visible reflectance to high NIR reflectance).

### 2.1. Visible Light Imaging

#### 2.1.1. Basic Principles

A visible image is based on digital images and is intended to mimic human perception to provide information or input to systems that need data for plant phenotyping applications to trait-based physiological breeding. The most common application of the visible image is based on silicon sensors (CCD or CMOS arrays) that are sensitive to visible bands of light (400–750 nm) and allow imaging in two dimensions, and it is the simplest imaging technology for plant sensing. Typically, the raw data of an image is presented in spatial matrices of intensity values corresponding to photon fluxes in the red (∼600 nm), green (∼550 nm), and blue (∼450 nm) spectral bands of visible light. Visible band cameras are commonly conventional digital cameras or RGB/CIR cameras because they can provide rapid measurements with affordable solutions for plant phenotyping applications.

#### 2.1.2. Current Applications

Visible images have been widely used in plant science for its low cost and its ease of operation and maintenance. In a controlled environment (in a growth chamber or in the greenhouse), visible imaging was primarily adopted for analyzing the shoot biomass [13,15], yield traits [18], panicle traits [44], imbibition and germination rates [46], leaf morphology [49], seedling vigor [23,32], coleoptile length and biomass at anthesis [93], seed morphology [42,94] and root architecture [11,45]. In one example for shoot biomass in a controlled environment, the projected leaf area in plants such as *Arabidopsis thaliana* and maize is available through commercial systems [7,24] that are based on visible imaging. This system used multiple viewing angles (usually two side views and a top view) to extract a mathematical relation between these three visible images for the shoot biomass or leaf area. The correlation between the digital estimation of the shoot biomass and that obtained for destructive harvest can exhibit an r^2^ value of greater than 0.9 [27,95].

In a controlled environment, the visible imaging of growth over a period of plant development can be used to estimate the sum of stress response mechanisms and offers the opportunity to tease apart many of these responses. Shortly after the application of salt stress, just as the stomata close, the inhibition of plant growth also occurs rapidly. After longer salinity exposure, leaf senescence can be quantified by separating the yellow and green areas of the leaf, and this finding can be related to tissue tolerance to accumulated salt. In using a visible image analysis, these components of salinity tolerance can be measured on a single plant. Furthermore, these components can be measured rapidly and accurately; thus, they can be measured in large populations such as mutant populations and mapping populations, which enables a genetic approach to be undertaken to identify genes, which underlies the variation in these respective components of tolerance [13,95,96].

In using the visible imaging extract of plant growth morphology dynamics, root systems, or seed surface features, a series of standard image analysis preprocessing and segmentation algorithms are used, such as the watershed algorithm, a color segmentation method based on RGB space and an image segmentation model [15]. In addition, new image processing methods were found. For example, De Vylder *et al.* [97] used color segmentation based on phenotype parameters extracted from RGB space including the shoot biomass (to quantify the image-based projected area), diameter (the maximum distance between two pixels belonging to the rosette), stockiness, relative growth rate, and compactness (the ratio between the area of the rosette and the area enclosed by the convex hull of the rosette). Massimo *et al.* [98] proposed a segmentation method for plants in image-based phenotyping experiments. The researchers built a plant appearance model based on Gaussian mixture models and prior knowledge. Using several top-view images of *Arabidopsis* that were collected using a time-lapse digital camera in the laboratory over a span of a few days, the proposed approach achieved an overall accuracy of 96.44%.

In the field, visible images provide information on the canopy cover and canopy color [99–101]. A canopy cover can be estimated by an image processing program of the color threshold. The leaf area index (LAI) and light interception [102] can be obtained with this method. Other more sophisticated information can also be extracted by image analysis such as water stress or salinity stress from the shape, compactness, and solidity [103]. In the field, a stereo camera rig or images from multiple locations [38] also allow for the detailed reconstruction of the canopy structure and its analysis to obtain critical variables for phenotyping, such as the LAI, leaf area distribution or panicle length [104].

#### 2.1.3. Advantages and Limitations

The traits named above are now scored manually, and this method can be improved in terms of both speed and accuracy by using visible imaging. Visible imaging in plant phenotyping is the simplest method, but these images can only provide plant physiological information in plant phenotyping. When visible images are processed to obtain phenotypic information such as the biomass, leaf number, and leaf area, it remains challenging to control the overlap of adjacent leaves in image segmentation. With the exception of this one difficulty, applications in the field are limited by the following conditions: (1) less difference in the brightness and color between the leaf and the background; (2) shadow removal of the canopy; (3) automatic fill when soil or insects are removed from the leaves; and (4) the influence of light on automatic image processing. These factors seriously affect the application of visible imaging to plant phenotyping in the field and must be solved by some other techniques.

### 2.2. Fluorescence Imaging

#### 2.2.1. Basic Principles

**Figure 3.**
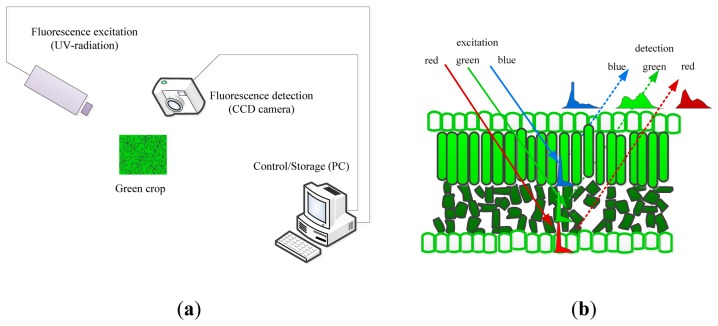
A scheme for the multi-color fluorescence imaging system (**a**) and the chlorophyll fluorescence emission of green leaves as induced blue, red and green excitation light (**b**) [109].

Information about a plant's metabolic status can be obtained by the artificial excitation of the plant photosystems and observation of the relevant responses. The most relevant technique to describe is its fluorescence. Fluorescence is light that is emitted during the absorption of radiation in some shorter wavelengths. The typically fluorescing part of the plant is the chlorophyll complex. Irradiating the chloroplasts with blue or actinic light will result in some re-emission of the absorbed light by the chlorophyll and the re-emission proportion of the absorbed light by the chlorophyll. The proportion of re-emission light compared with the irradiation is variable and depends on the plant's ability to metabolize the harvested light. This re-emitted light is the fluorescence, and it is a good indicator of the plant's capacity to assimilate actinic light. Moreover, combining an actinic light source with brief saturating blue pulses may be used to estimate the plant's efficiency of photo-assimilation, non-photochemical quenching and other physiological plant parameters. Fluorescence imaging is the imaging of these fluorescence signals (or parameters) and generally employs charge-coupled device (CCD) cameras that are sensitive to fluorescence signals where the fluorescence signals occur by illuminating samples with visible or UV (ultraviolet ) light by means of pulsed lasers, pulsed flashlight lamps or LEDs (light emitting diodes) as shown in Figure 3a. Pixel value images of the fluorescence parameters were displayed with the help of a false color code ranging from black (0.00) through red, yellow, green, and blue to pink (ending at 1.000) [105,106].

UV (ultraviolet) illumination (ranging from 340–360 nm) generates two types of fluorescence, that is, the red to far-red region and the blue to green region, which is the principle underlying multicolor fluorescence imaging. This technique permits the simultaneous capture of fluorescence emission from four spectral bands (blue (440 nm, F440), green (520 nm, F520), red (690 nm, F690) and far-red (740 nm, 740)) by excitation with a single wavelength. The emission of fluorescence signals of blue and green origin (with maxima near 440 and 520 nm) are the cinnamic acids (primarily ferulic acid) present mostly in the cell walls, and the origins of red and far-red fluorescence emission (with maxima near 690 and 740 nm) are chlorophyll α molecules in the antenna and reaction center of the photosynthetic photosystem II from chloroplasts in the mesophyll cells (as shown in Figure 3b [107]). Changes in the fluorescence emission or, even more sensitive, changes in the relation between different fluorescence ratios (F440/F690, F440/F735) may be used as indicators of stress, and the F690/F735 ratio has been shown to be an indicator of the chloroplast content [106–108].

#### 2.2.2. Current Applications

The primary technique used for disease detection in leaves is fluorescence imaging. During disease infection, metabolic changes occurring from photosynthesis to respiration and during nutrient flow derivation are the first to be affected. This process is primarily monitored by fluorescence imaging. Because using modulated fluorescence requires substantial power for rapid illumination, fluorescence imaging is often used in a controlled environment.

Fluorescence imaging can estimate photosynthesis to monitor the effects of plant pathogens [21,110] and diagnose early stress responses to abiotic and biotic factors before a decline in growth can be measured [52,59,111–113]. In plant phenotyping, fluorescence imaging is primarily used to image other physiological phenomena indirectly if they interfere with the operation of photosynthesis and its associated metabolism, e.g., herbicide effects and stomatal heterogeneity for screening genotypes with disease resistance, and for those that are tolerant to abiotic and biotic stress. A few examples of their applications to plant phenotyping are given.

Fluorescence imaging provides a rapid screening technique to identify plants with improved or impaired metabolism and growth. Perturbations in metabolic processes (as caused by pathogens and stress) that are not directly involved in photosynthetic metabolism often induce changes in fluorescence parameters, which can be used to screen for such perturbations. Barbagallo *et al.* [109] used images of the fluorescence parameter *F_v_*/*F_m_* for seedlings growing in a 96-well plate, and the results showed that a range of Imazapyr treatments produced a marked decrease in this parameter after 24 h, with a magnitude of decrease related to the concentration of herbicide that was applied.

To screen genotypes, fluorescence imaging has been proposed as a tool for the study of stress-induced compounds that could be screened as indicators of stress responses. For example, Swarbrick *et al.* [57] used the quantitative imaging of chlorophyll fluorescence to study the resistance response of barley leaves infected with *Blumeria graminis*. During a susceptible interaction, photosynthesis was progressively reduced across the whole leaf. Chaerle *et al.* [59] screened sugar beet lines that differed in their susceptibility to *Cercospora beticola* infection by using chlorophyll fluorescence imaging and showed that differences in fluorescence intensity were measured between susceptible and resistant plants. Burling *et al.* [51] studied differences in the level of wheat cultivar resistance in response to *Puccina triticina* using fluorescence imaging and showed that they can be discriminated using the quantum yield of non-regulated energy dissipation in PSII. For a susceptible cultivar, a more pronounced difference between parameter values was measured in the control and inoculated leaves and as a distinct evolution over time.

In addition, several possible uses of chlorophyll fluorescence imaging have been proposed for mapping quantitative trait loci (QTLs) for growth-related traits [114,115] (such as the leaf area) by high-throughput screening for photosynthetic mutants or transformants and by characterizing mutants with different photosynthetic pigment compositions [51,57,116,117]. To take measurements at the whole plant and canopy level, methods that use laser-induced fluorescence transients [118] or the monitoring of sun-induced fluorescence [119] are explored.

#### 2.2.3. Advantages and Limitations

The imaging of chlorophyll fluorescence provides a powerful tool to resolve the spatial heterogeneity of leaf photosynthetic performance, and it has been used in many areas of plant physiology such as the early detection of stress symptoms induced by pathogen attack or herbicide treatment [51,57,116]. Some measures such as growth differences cannot be detected by visual observation, and there are very large differences in the images between the control and herbicide-treated plants [109].

However, the ratio of variable and maximum fluorescence that was measured after saturating light pulses appears to be relatively insensitive to severe water limitation [14], and thus it does not seem suitable for the early detection of water stress. Most fluorescence imaging studies are limited at the level of single leaves or the seedling level of model crops. Robustness, reproducibility and data analysis software are needed to address the use of large scale phenotyping and to develop a standard procedure for fluorescence image processing. In addition, the power requirements of fluorescence imaging (for example, using short-wave laser stimulation) may be limiting for field phenotyping applications.

### 2.3. Thermal Imaging

#### 2.3.1. Basic Principles

Thermal imaging allows for the visualization of infrared radiation, indicating an object as the temperature across the object's surface. The sensitive spectral range of thermal cameras is 3–14 μm, and the most commonly used wavelengths for thermal imaging are 3–5 μm or 7–14 μm. Within wavelengths of these two ranges, infrared radiation atmospheric transmission is close to its maximum value. The thermal sensitivity of smaller wavelengths is 3–5 μm, which makes it higher than that of wavelengths at 7–14 μm because small wavelengths correspond to higher energy levels. However, the use of longer wavelengths may be advantageous for certain applications. For example, for targets at a long distance through longer atmospheric paths, wavelengths between 8 and 14 μm would minimize errors from the atmospheric absorption of infrared radiation [120,121]. In recent years, given the development of infrared thermal technology, thermal cameras with very high thermal sensitivity (of some milliKelvins) were made available [121] and readily revealed temperature distributions at the plant canopy to leaf level, which tends to further lower their price, and they have a more user friendly interface and increased availability of higher resolution detectors.

#### 2.3.2. Current Applications

Thermal imaging is used to measure leaf surface temperatures to study plant water relations, and specifically for stomatal conductance, because a major determinant of the leaf temperature is the rate of evaporation or transpiration from a leaf. Abiotic or biotic stresses often result in decreased rates of photosynthesis and transpiration [122,123]; and, the remote sensing of the leaf temperature by thermal imaging can be a reliable way to detect changes in the physiological status of plants in response to different biotic and/or abiotic stresses. The canopy temperature has been used successfully in breeding programs for drought-prone environments. In plant phenotyping, thermal imaging offers canopy temperatures to detect differences in stomatal conductance as a measure of the plant response to the water status and transpiration rate [56,64], both in the field and in the greenhouse. In a study conducted by Giuseppe, thermal infrared imaging was used to distinguish among 92 different maize genotypes for screening drought adaptation in maize. There was a mean temperature difference of more than 2 °C between different genotypes under water stress [124]. Romano *et al.* [125] used thermal imaging at the canopy level in maize under reproductive stage drought stress between anthesis and the blister stage. Thermal imaging was identified as a potential tool that can accelerate phenotyping and screening in maize water stress breeding programs. Thermal infrared imaging has also been proposed for use in the lab for mutant screens in *Arabidopsis* [126]. Canopy temperature differences were compared with the surrounding air (for example, the canopy temperature depression, or CTD) as measured by thermal infrared imaging, and these results have been used as a selection criterion in breeding programs for drought resistance [71]. The thermal imagery of a single cover crop under drought stress has been used to identify within-field variability for phenotyping site selection.

In addition, thermal imaging has been used for many crops, from small cereal grains to maize [127] and fruit trees [128]. It has also been used in combination with spectral imaging for the enhanced estimation of leaf water content [125].

#### 2.3.3. Advantages and Limitations

Although thermal cameras are more expensive and more difficult to handle than infrared sensors, thermal cameras offer several benefits, such as spatial resolution and more precise measurements under changing environmental conditions. Furthermore, a large number of plots in field trials can be imaged at the same time, ideally allowing for a comparison of differences in the canopy temperature among genotypes without the need for normalization to determine the absolute leaf temperature. However, plant thermal analysis based on thermal infrared imaging is influenced greatly by being around any object and environment. Surface temperature measurements require extensive calibration. In addition, if the plants with more complex three-dimensional morphology are studied, both the orientation of the leaves towards the incident radiation and the camera angle used to record the images must be considered in the data analysis [64].

In addition, thermal images, in conjunction with visible and NIR images, enable the exclusion of non-leaf material when estimating the canopy temperature and the possibility of selecting specific parts of the canopy for water stress estimation [129–131]. Spatial patterns are created based on one of the images (e.g., the color processing of the RGB image) and then superimposed on the other (e.g., a mask on the thermal image). This approach allows for the isolation of leaves that are exposed to uniform environmental conditions and enables a better interpretation of their temperature according to known prevailing environmental conditions.

### 2.4. Imaging Spectroscopy

#### 2.4.1. Basic Principles

The application of imaging spectroscopy to plant phenotyping came from research on the remote sensing of vegetation. Plant imaging spectroscopy is performed using the interaction of solar radiation produced with plants. In the visible spectrum (400–700 nm), reflectance by single leaves or canopies is particularly low. This low reflectance is explained by the absorption by leaf pigments, primarily chlorophyll, with a characteristic peak of reflectance in the green region of approximately 550 nm. With the transition from the visible to near infrared (NIR) wavelengths, there is a sharp increase in reflectance, or the so-called ‘red edge’. In the NIR (700–1200 nm), a large proportion of incident radiation is reflected by leaves from scattering within the leaf mesophyll. Furthermore, NIR radiation can be transmitted from the upper leaves of the canopy to the lower leaves, which can reflect the photons back to the upper part of the canopy. As a consequence, leaf and canopy architecture, such as leaf thickness and growth habit, are the major determinants of the reflectance pattern in this part of the spectrum. With increasing wavelengths of up to 2500 nm, the reflectance decreases gradually because of increased absorption by the water present in the leaves [33,132]. Near infrared spectroscopy for the indirect assessment of crop growth and yield performance under potential yield and stress conditions has been addressed more recently [133]. Spectral reflectance information from leaves or canopies is used to quantify vegetation indices, which are simple operations (e.g., ratios and differences) between spectral reflectance data at given wavelengths. This finding enabled the development of the normalized difference vegetation index (NDIX) and a wide range of related indices. These vegetation indices are usually related to different plant characteristics such as the photosynthetic active biomass, pigment content and water status [92]. Vegetation indices have been used to predict the green biomass, leaf area, chlorophyll content and yield in wheat and maize under field conditions [134,135].

Both near infrared spectroscopy and plant spectral reflectance rely on the development of calibration models that relate the spectral information and reference data of the trait. Usually, a sub-sample from a complete data set representing the entire population in terms of the range of spectral variation is used for calibration development with the appropriate mathematical treatments and algorithms to build robust prediction models. These appropriate mathematical treatments and algorithms include 2D correlation plots [136], partial least squares regression [137,138], principal components analysis [139], support vector machines [140], neural networks [141] and other machine learning approaches. Once the calibration models have been successfully validated, they can later be employed in routine analyses to predict phenotypic values on external data sets by using spectral data and further used in combination with environmental and genotypic data to make breeding decisions [142,143].

Spectral measurement can be obtained by multispectral or hyperspectral imaging cameras that are capable of scanning wavebands of interest at high resolutions.

#### 2.4.2. Current Applications

In plant phenotyping, spectral reflectance indices are used for fast, non-destructive measurements of green biomass, canopy chlorophyll content, leaf and canopy senescence (or if they stay green) and plant water status. The derivation of a number of reflectance vegetation indices, from simple differences between two wavelength reflectance values to normalized reflectance values, is often used. Several indices have been introduced in both field research and breeding programs for large-scale phenotyping and dynamic estimations of the biomass, greenness, nitrogen content pigment composition, photosynthetic status, and water content [144–146].

Multispectral and hyperspectral measurements are widely used to estimate the canopy water content as an indicator of water status, which uses the absorption bands in the infrared range to describe various water indices [100,147–149]. Moreover, the use of high resolution spectroscopy and wavelet analysis can also provide high sensitivity to the canopy water content [150,151]. The high spectral resolution hyperspectral measurement makes it a promising method for assessing rice leaf growth [73], for determining the condition of rice panicles [74] and for detecting the severity of damage caused by insects [152–154], such as the results of an investigation by Sabatier *et al.* [155] using near infrared reflectance spectroscopy as a high-throughput screening tool for pest and disease resistance in a sugarcane breeding program. Near infrared reflectance spectroscopy predicted the constitutive components of resistance to pests and diseases in germplasms from the South African sugarcane breeding program. Two hundred and twenty-two genotypes were scanned over the 1100–2300 nm wavelength range by a fiber-optic probe. Partial least square (PLS) regressions were applied to bud, internode and leaf spectra that were pretreated (second derivative) and scatter-corrected (SNV and detrending). Calibration models resulting from the correlations between NIR measurements and existing ratings gave coefficients of determination for calibration (r^2^, the closer to one the better) and standard errors of prediction by leverage correction (SEP, the lower the better) of 0.72 (SEP 1.19) for the African stalk borer (*Eldana saccharina*), 0.62 (SEP 1.50) for smut (*Sporisorium scitamineum*), 0.62 (SEP 1.07) for sugarcane thrips (*Fulmekiola serrata*) and 0.67 (SEP 1.02) for brown rust (*Puccinia melanocephala*) ratings. Apoorva *et al.* [156] evaluated vegetation indices for the precision phenotyping of quantitative stripe rust reactions in wheat. One hundred and twenty Indian wheat genotypes were scanned using an optical handheld GreenSeeker sensor to record the normalized difference vegetation index (NDVI) and a handheld plant chlorophyll meter measured the leaf chlorophyll content. The results indicate that temporal ground-based NDVI is most effective for studying the quantitative rust reaction with a significant regression coefficient (r^2^ = 0.63) between the area under the disease progress curve and NDVI data as followed by the chlorophyll content index (r^2^ = 0.37).

For plant phenotyping, an investigator looked into the possibility of using specific bands in the NIR to the mid-infrared region to estimate tissue water content noninvasively and to design screening protocols for genotypic differential responses to drought [64,157,158]. For example, Cabrera *et al.* [159] used near infrared spectroscopy to accurately predict genotypic differences in the kernel and leaf ash content and nitrogen in maize grown under different water treatments. Near infrared spectroscopy has also been proposed to predict isotopic signatures associated with genotypic adaptation to water stress such as the stable isotope composition of carbon [160] and oxygen [159] in mature kernels. In further extending the number of measured wavelengths, imaging spectroscopy opens new possibilities for extracting spectral features related to plant health and disease status.

#### 2.4.3. Advantages and Limitations

The use of near infrared spectroscopy and spectral reflectance techniques for plant phenotyping is very promising. Its applications in their infancy are mature and reliable. When combined with aerial platforms (such as helicopters, balloons and cranes), it is very well-suited for field phenotyping.

At present, the limit of employing imaging spectroscopy to high-throughput screening applications is the large volume of data that can be generated from spectral images. In addition, the costs of multispectral or hyperspectral imaging cameras are relatively expensive, which among other reasons, prevents their wide adoption by breeding programs.

### 2.5. Other Imaging Techniques

#### 2.5.1. Some Techniques for the 3D Mapping of Plants

The available imaging sensor technologies used for the 3D mapping of plants at present are primarily light detection and ranging (LIDAR) (or laser scanner) sensors [161], stereo vision [39,82], photon mixer devices (PMD) time-of-flight cameras [79] and even consumer-gaming interface Microsoft Kinect [162,163]. With its greater robustness, accuracy and resolution, the best known and most widely used type of sensor for 3D canopy reconstruction is LIDAR [164–166]. It creates accurate and detailed 3D models by structured light projection and laser range scanners. However, it can be expensive, complex and require longer imaging times. Laser scanners have been used for rapid LAI mapping [167] and for estimating the plant area density profiles of a wheat canopy [168]. Light detection and ranging (LIDAR) (or laser scanner) is an active remote sensing technique that uses liar sensors to measure the 3D distribution of plant canopies directly. After being processed further for geometric structures associated with plant organs, 3D data from LIDAR methods can provide high-resolution topographic maps and highly accurate estimates of vegetation high, cover, and canopy structures [161,168]. Furthermore, when combined with fluorescence, laser scanning enables the evaluation of photosynthetic performance and has potential in areas such as plant pathology [169].

Stereo vision has two or more cameras or structures from motion techniques for 3D data. However, stereo correspondence and depth accuracy vary with the type of algorithm used. Local correspondence algorithms are efficient but less accurate than global ones. Moreover, the performance is adversely affected by the lack of surface texture on the object. Stereo vision has been successfully used indoors; for example, Mizuno *et al.* [170] used stereo vision for wilt detection and Takizawa *et al.* [171] used stereo vision to construct 3D models of plants. From these models, the information extracted such as the plant height, leaf area and shapes are helpful in plant phenotyping. In field operations, it has been successful for imaging at larger scales, such as when Rovira *et al.* [172] used aerial stereo images for growth estimation. The use of stereo vision for corn plant space sensing both indoors and outdoors has been demonstrated by Jin *et al.* [173]. To some extent, taking these structural measurements with stereo vision has been attempted outdoors. Ivanove *et al.* [174] used top stereo images of maize plants in the field to find structural parameters such as the leaf orientation, leaf area distribution and leaf position to then construct a canopy model. After performing a destructive analysis of the plant to view the inner leaves, the 3D model properties were not promising. However, the methods and imaging apparatus have improved greatly since then. In addition to the texture, sunlight is also an important factor affecting stereo vision performance. To avoid sunlight, either a shade is used or experiments are performed on overcast days [175]. Strong sunlight and the stereo matching process reduce the efficacy of stereo vision and limit either the scope or the scale of the application.

Recent advances in the Time of Flight based on range sensors have revolutionized the industry, and several brands of off-the-shelf 3D cameras are available in the market. These cameras employ near infrared emitters and generally produce low resolution depth images. Their resolution has been gradually increasing over the last few years. They can produce a high frame rate (up to 50 fps) and depth of images and are therefore highly suitable for real-time applications. However, their performance is affected by sunlight [176]. In comparison with stereo vision and LIDAR, fewer ToF camera applications for plant phenotyping have appeared. However, they are costly in relation to conventional 3D systems. Kraft *et al.* [177] and Klose *et al.* [79] investigated the feasibility of ToF cameras for plant analysis. They found it to be a good candidate for plant phenotyping but they failed to account for the IT (IT: a controllable parameter related to the length of time the sensor integrates the returned signal), which is a very important parameter, and without it, ToF data evaluation becomes somewhat meaningless. Alenya *et al.* [178] used a ToF camera indoors by combining depth data with RGB images for leaves. Going a step further, Song *et al.* [179] combined ToF images with stereo images for plant leaf area measurements in a greenhouse to increase the resolution for the depth data. A ToF camera was used in corn fields for inter-plant space measurement [180]. Wind and sunlight were blocked from view using a shade. Low resolution and sensitivity to outdoor illumination have become two major challenges for ToF applications.

Recently, the low cost Microsoft Kinect has been used to research phenotyping [82,162]. This instrument has low resolution and is highly sensitive to outdoor lighting, limiting its application.

#### 2.5.2. Tomographic Imaging by MRI, PET or CT

MRI (Nuclear Magnetic Resonance Imaging) employs nuclear magnetic resonance to generate images and detects nuclear resonance signals originating from ^1^H, ^13^C, ^14^N and ^15^N. An MRI can acquire 3D datasets of plant structures and be used in seeds [181], complete root systems growing in or near natural soil [182] and entire plants [183]. In addition, MRI can describe 3D representations of water distribution and be applied for the noninvasive quantification of plants or plant organ water content and to estimate water diffusion and water transport [184], and for uncovering labeled molecules [181].

PET (Positron emission tomography) is a nuclear imaging technique that produces a 3D image or picture of a functional process. It detects pairs of gamma rays that are emitted indirectly by a positron-emitting radionuclide. It can noninvasively image the distribution of labeled compounds, such as ^11^C [185], ^13^N [186], or ^52^Fe [187]. When CO_2_ is consumed during photosynthesis, the transport of ^11^C-labeled photo-assimilates can be repeatedly imaged in 3D by PET. This imaging mode can dissect transport domains in plant organs, and it delivers quantitative parameters such as transport velocities and the lateral loss rate along transport paths [188]. When used in combination with MRI, it can provide structural and functional traits and can be used to analyze the transport of water and labeled compounds independently.

X-ray CT (X-ray computed tomography) is a technology that uses computer-processed X-rays to produce tomographic images of specific areas of the scanned object and can generate a 3D image of the inside of an object from a large series of 2D radiographic images taken around a single axis of rotation. This technique can provide volumetric data for various structures with different densities such as soil structural heterogeneity [189] and plant structures [190], and it can measure root system architecture (a bias of 8%) [10]. In root system architecture systems, the CT method has been applied to a number of species including barley, maize, *Arabidopsis*, wheat, and chickpea [191–195]. The limitations of CT are its cost and scanning times.

However, the above tomographic imaging technologies remain low throughput and their image segmentation and reconstruction must be further improved for high throughput plant phenotyping.

As described above, although the individual imaging techniques are able to reveal symptoms at early stages for a wide range of stresses, effective discrimination between causal stresses is improved by the use of multiple sensors (such as thermal imaging and fluorescence imaging) that monitor different physiological processes. For example, both water stress and nitrogen deficiency can reduce the chlorophyll concentration (which is revealed by changes in fluorescence imaging), but water stress typically has a more pronounced and faster effect on stomatal closure (as detected by thermal imaging), given that only water stress leads to leaf wilting. Water stress will also inhibit photosynthesis by the stomatal limitation of CO_2_ uptake, which will affect chlorophyll fluorescence emission. The kinetics of chlorophyll fluorescence emission will likely differ between water stress and nitrogen deficiency [196]. To visualize the dynamics of stomatal patchiness to interpret the heterogeneity of stomatal and possibly linked photosynthetic responses, it is necessary to have multi-sensor fusion-phenotyping platforms [197].

## 3. Examples of Phenotyping Platforms

By combining advances in sensing technologies, automatic control technology, and aeronautics, computing is paving the way to developing controlled environment-based phenotyping platforms and field-based platforms (shown in Table 3). Recently, controlled environment-based phenotyping platforms have been sold commercially or developed in the public domain and have been deployed in growth chambers or greenhouses. These platforms are specifically designed for research and large scale phenotyping for a limited range of species, encompassing small rosette plants such as *Arabidopsis* [15,27] and the primary cereal crops [13,29,198]. Much of the discussion of these platforms has focused on the intensive measurement of individual plants by using platforms that combine robotics and image analysis with controlled environment systems [15]. However, the use of controlled environments to represent field environments has well-known limitations. Limited greenhouse space or chamber volumes often do not allow plants to flower and set seed, making it impossible to assess the effects of stresses during reproductive growth. The soil volume that is provided for plants in controlled environments is usually far less than that available to plants in the field, affecting nutrient and water regimes and altering normal patterns of growth and development. Enclosed controlled environments are also problematic for characterizing responses relevant to field environments. For example, greenhouses or chambers, solar radiation, wind speed and evaporation rates are typically lower than they are under open-air situations. Researchers have focused on field-level improvements in yield potential or abiotic stress tolerance that favors field-based phenotyping. Field-based phenotyping platforms are increasingly recognized as the only tool for delivering the requisite throughput in terms of the numbers of plants or populations and an accurate description of trait expression in the real world [31].

The field-based platforms include ground-based and aerial-based methods. Ground-based phenotyping platforms include modified vehicles and sensing sensors, which are often referred to as ‘phenomobiles’. Different ‘phenomobiles’ have been developed within the past few years [31,78,199–202]. For example, a triticale has been developed that carries eight sets of sensors, two 3D Time of Flight cameras, a color camera, three laser distance sensors, a hyperspectral imaging system and two light curtain imaging systems to measure the plant height, fresh weight density, moisture content, growth stage, tiller density and nitrogen content of all plots, enabling the screening of approximately 250 plots per hour [78]. Ground-based phenotyping platforms enable the data to be captured at the plot level and require little post-processing. However, this platform also limits the scale at which ground-based phenotyping platforms can be used. Furthermore, simultaneous measurements of all plots within a trial are not possible with ground-based phenotyping platforms.

Aerial-based phenotyping platforms are increasingly being considered as an alternative option to overcome limitations associated with ground-based phenotyping platforms. Aerial-based phenotyping platforms enable the rapid characterization of many plots within minutes. Initial aerial-based phenotyping platforms used small airplanes (e.g., crop-dusting airplanes); however, this is costly and it is difficult to safely achieve the low speeds required for high-definition images at low altitude. The current generation of aerial-based phenotyping platforms significantly varies in terms of the payload, initial costs, maintenance costs, and control. Recently developed alternatives include phenotowers and blimps. However, these types of aerial-based phenotyping platforms have a maximum height of 50 m. Blimps are helium-filled balloons that can be held at an appropriate position and have sensors mounted underneath. Their advantages are their ability to carry a heavy payload (several kilograms), and they can make many sensors work concurrently; but they need many people to control the considerable room for storage upon inflation. Unmanned aerial platforms are alternatives to blimps, for example polycopters [203,204] and airplanes [205]. In comparison with blimps, the payload of unmanned aerial platforms is lower, but these vehicles can generally carry up to 2 kg, and they can have at least two sensors mount for simultaneous imaging. Unmanned aerial platforms have greater flight control and autonomy and are becoming increasingly affordable. Although polycopters can be maneuvered into an appropriate position, the unmanned airplanes relying on advances in aeronautics and sensors can obtain high-quality images. The autonomy and area covered by airplanes are larger and the risks of destruction by craning are lower than for polycopters. Most unmanned aerial platforms carry an RGB/CIR camera and thermal imaging sensor. Alternatives to the RGB/CIR cameras are multispectral or hyperspectral imaging sensors, although they increase the payload but bring a huge range of new possibilities. For unmanned aerial platforms, the software includes programs to do the following: (1) plan fight missions; (2) gather the images; and (3) extract the data for plots within the images. An imager is operated from the ground station. The ground station controls the unmanned aerial platforms by radio link, transmitting the position, altitude and status [40,41].

Multifunctional platforms that obtain a large quantity of images and data make high capacity computing and data storage essential in software for phenotyping platforms. Analyzing and managing these data pose another informatics challenge as well, in particular, when the projects become large in scale and many people are involved. Because a single image has the potential to yield a large number of measurements or phenotype descriptions, these factors add further to the complexity of the subsequent data analysis. Billiau *et al.* [206] faced this problem and described how they solved the challenge. A laboratory information management system was already in place and was augmented with another shell of programs that handled data storage, retrieval and accessibility. As such, safe access and fast data handling could be achieved, ensuring fast data handling, and access to all scientists and stakeholders in the project [207].

## 4. Conclusions

In this review, we have assessed a range of different wavelength imaging techniques in plant phenotyping (Table 1). For the imaging sensors applied to plant phenotyping, physical properties, depth knowledge, robust software, and image analysis pipelines are prerequisites to enable the collection of phenotype data. Visible imaging for the estimation of shoot biomass and growth patterns in 2D (individual leaves to canopies) has been used reliably for crops in breeding. Fluorescence imaging was primarily used for foliar disease detection and thermal imaging for plant water status detection. A 3D surface reconstruction requires calibration for biomass estimation. Imaging spectroscopy requires standard procedures for the extraction of spectral features to reduce raw data in plant phenotyping. For the MRI and PET of plant phenotyping in screening, data acquisition is time consuming, and software tools must be further developed to analyze data and obtain physiologically interpretable results. There is a large difference in the reliability of imaging methods between controlled environments and the field (shown in Table 2). This reliability must be considered to understand the measurement principle for each experimental design, proper sensor calibration, and regular calibration of the imaging-based systems.

In addition to the techniques reviewed, there are other technologies that have led to significant contributions to a plant phenotyping level of understanding about plant responses in heterogeneous environments, such as a microwave resonator prototype [12] and light curtain imaging (LCI) [78,200]. The microwave resonator phenotype can estimate plant biomass dynamically at various spatial and temporal resolutions, and light curtain imaging can obtain plant contours under different light environments. With the refinement of current imaging technologies and the development of new techniques, more information will be available to help dissect plant phenotypes and speed up plant phenotyping.

## Figures and Tables

**Table 1.** A comparison of different imaging techniques in plant phenotype application [23,40,41].

**Imaging Techniques**	**Sensor**	**Resolution**	**Raw Data**	**Phenotype Parameters**	**Examples of Species**	**Imaging Environment**
Visible light imaging	Cameras sensitive in the visible spectral range	whole organs or organ parts, time series	Gray or color value images (RGB channels)	Projected area, Growth dynamics, Shoot biomass, Yield traits, Panicle traits, Root architecture, Imbibition and germination rates, Early embryonic axis growth, Height, Size morphology, Flowering time	*Arabidopsis thaliana* [15,27,42,43]; Barley [13]; Rice [11,18,44,45]; Legume *Medicago truncatula* [46]; Maize [47,48]; Bean [49]	Controlled environment; field
Fluorescence imaging	Fluorescence cameras and setups	Whole shoot or leaf tissue, time series	Pixel-based map of emitted fluorescence in the red and far-red region	Photosynthetic status (variable fluorescence), quantum yield, non-photochemical quenching, leaf health status, shoot architecture	Wheat [50,51]; *Arabidopsis* [14,52–54]; Natural grassland, winter wheat, corn [55]; Barley[56,57]; Bean[58]; Sugar beet [59]; Tomato [60]; Chicory plant [61];	Controlled environment; Field
Thermal imaging	Near-infrared cameras,	Pixel-based map of Surface temperature in the infrared region	Whole shoot or leaf tissue, time series	Canopy or leaf temperature, insect infestation of grain	Barley [56]; Wheat [56,62]; Maize [63]; Grapevine [64]; Rice [64];	Controlled environment; Field
Near infrared imaging	Near-infrared cameras, multispectral line scanning cameras, active thermography	Continuous or discrete spectra for each pixel in the near-infrared region	Time series or single-time-point analyses of shoots and canopies, single-point assessment of seeds	water content composition parameters for seeds, leaf area index	Rice[65–67]; Soybean [68]; Maize [69,70]; Barley [71]; Wheat [56]	Controlled environment
Hyperspectral imaging	Near-infrared instruments, spectrometers ,hyper spectral cameras, thermal cameras	Crop vegetation cycles, indoor time series experiments	Continuous or discrete spectra	Leaf and canopy water status; Leaf and canopy health status; panicle health status; leaf growth; Coverage density	Rice [72–76]; Wheat [50]; *Arabidopsis* [77]; Triticale [78]	Field; Controlled environment
3D imaging	Stereo camera systems; time-of-flight cameras	Whole-shoot time series at various resolutions	Depth maps	Shoot structure; leaf angle distributions; canopy structure; root architecture; Height	Soybean [39]; Rosebush; Maize [79]; Triticale [78]; Pepper [80]	Field; Controlled environment
Laser imaging	Laser scanning instruments with widely different ranges	Whole-shoot time series at various resolutions	Depth maps, 3D point clouds	Shoot biomass and Structure; leaf angle distributions; canopy structure; Root architecture; Height; Stem	Maize [81]; Sugar beet and wheat ears [82]; Triticale [78]; Barley [83]; Soybean [84];	Field; Controlled environment
MRI	Magnetic resonance imagers	200–500 μm; 1–600 s	Water(^1^H) mapping	Morphometric parameters in 3D; Water content	Sugar beet [85]; *Hordeum spontaneum* and *Beta vulgaris* [86]; Bean [87]	Controlled environment
PET	Positron emission detectors for short-lived isotopes (e.g., ^11^CO_2_)	1–2 mm; 10 s–20 min	Radiotracer mapping and coregistration with positron emission signals	Transport partitioning, sectorality, flow velocity	*Hordeum spontaneum* and *Beta vulgaris* [86]	Controlled environment
CT	X-ray computed tomography and X-ray digital radiography	100 μm and lower; hours	Voxels and tissue slices	Tillers; Morphometric parameters in 3D; grain quality	Rice [88]; Wheat [10,89–91]	Controlled environment

**Table 2.** The application and limitations of imaging techniques for plant phenotyping under different growing environments [23,41].

**Imaging Techniques**	**Growing Environment**	**Applications**	**Limitations**
Visible imaging	Controlled environment	Growth dynamics, Shoot biomass, Yield traits, Panicle traits, Root architecture, Imbibition and germination rates, leaf morphology, seedling vigor, coleoptile length and biomass at anthesis, seed morphology, root architecture	Only provides plant physiological information
	Field	Imaging canopy cover and canopy colour; colour information can be used for green indices; the use of 3D stereo reconstruction from multiple cameras or viewpoints allows the estimation of canopy architecture parameters	No spectral calibration; Only relative measurement; shadows and sunlight can result in under or over exposure and limit automatically processing image
Fluorescence imaging	Controlled environment	Photosynthetic status, indirect measurement of biotic or abiotic	Difficult to analysis complicated whole-shoot of non-rosette species; pre-acclimation conditions required
	Field	Photosynthetic status, indirect measurement of biotic or abiotic stress	Difficult to measure at the canopy scale, because of the small signal to noise ratio, though laser-induced fluorescence transients can extend the range available, while soar-induced fluorescence can be used remotely
Thermal imaging	Controlled environment	Surface temperature; stomatal conductance water stress induced by biotic or abiotic factors	Imaging sensor calibration and atmospheric correction are often required; sound physics-based results interpretation needed
	Field	Stomatal conductance; water stress induced by biotic or abiotic factors	Imaging sensor calibration and atmospheric correction are often required; Changes in ambient conditions lead to changes in canopy temperature, making a comparison through time difficult, necessitating the use of reference. Difficult to separate soil temperature from plant temperature in sparse canopies, limiting the automation of image processing.
Imaging spectroscopy	Controlled environment	water content composition parameters for seeds; leaf area index; Leaf and canopy health status; panicle health status; leaf growth; Coverage density	Sensor calibration required; cost, large image data sets for hyperspectral imaging, complex data interpretation
	Field	Biochemical composition of the leaf or canopy; pigment concentration; water content; indirect measurement of biotic or abiotic stress; canopy architecture, LAI or NDVI	Sensor calibration required; changes in ambient light conditions influence signal and need frequent white reference calibration; canopy structure and camera geometries or sun angle influence signal. Data management is challenging
LIDAR	Controlled environment	Canopy height and canopy architecture; estimation of LAI; volume and biomass; reflectance from the laser can be used for retrieving spectral information	Specific illumination required for some laser scanning instruments
	Field	Canopy height and canopy architecture; estimation of LAI; volume and biomass; reflectance from the laser can be used for retrieving spectral information	Integration or synchronization with GPS and encoder position systems is required for georeferencing

**Table 3.** Relative advantages and disadvantages about typical phenotyping platforms [41].

**Phenotype Platform Type**	**Advantages**	**Disadvantages**
Controlled environment based	Automatically continuous operation; good repeatability	Generally expensive; can only monitor a very limited number of plots
Ground based	Very flexible deployment; good capacity for GPS/GIS tagging; very good spatial resolution	Generally take a long time to cover a field, so subject to varying environmental conditions
Aerial based	Can cover the whole experiment in a very short time, getting a snapshot of all of the plots without changes in environmental conditions	Limitations on the weight of the payload; spatial resolution depends on speed and altitude
